# The Influence of Alkali Metals on the Sintering Mineralization Process of Iron Ore

**DOI:** 10.3390/ma18020227

**Published:** 2025-01-07

**Authors:** Xintai Jiang, Fenglin Lu, Jiantao Ju, Wenke Guo, Jian Zu

**Affiliations:** 1 Gansu Jiugang Group Hongxing Iron and Steel Co., Ltd., Jiayuguan 735100, China; jiangxintai@jiugang.com (X.J.);; 2 School of Metallurgical Engineering, Xi’an University of Architecture and Technology, Xi’an 710055, China15309216845@163.com (J.Z.)

**Keywords:** alkali metal compounds, sintering basic characteristic, microhardness, first principles, thermodynamic

## Abstract

To investigate the influence of alkali metal compounds in different forms on the sintering mineralization process of iron ore, the basic sintering characteristics of iron ore with alkali metal contents ranging from 0 to 4% were measured using the micro-sintering method, and the influence mechanism was analyzed using thermodynamic analysis and first-principles calculations. The results showed that (1) the addition of KCl/NaCl increased the lowest assimilation temperature (LAT) and the index of liquid-phase fluidity (ILF), while that of K_2_CO_3_/Na_2_CO_3_ decreased the LAT but increased the ILF of iron ore. (2) The pores formed by the volatilization of KCl/NaCl suppressed the diffusion of Fe^3+^ and Ca^2+^, which inhibited the formation of silico-ferrite of calcium and aluminum (SFCA). The addition of K_2_CO_3_/Na_2_CO_3_ promoted the formation of a silicate liquid phase with better fluidity, intervening in the solid-phase reaction between iron ore and CaO. (3) The alkali metal compounds in different forms concentrated in silicate but showed lower levels of distribution in iron-bearing minerals in the form of a solid solution. Furthermore, the formation of an alkali metal-bearing solid solution decreased the microhardness of minerals. This decrease in microhardness and in the content of the SFCA bonding phase directly contributed to the decrease in the compressive strength of the sinter.

## 1. Introduction

Sinter is the primary iron-bearing feedstock of blast furnaces in China at present, so the quality and metallurgical properties of sinter directly affect the energy consumption and operation of a blast furnace [1,2]. In recent years, steel enterprises have used metallurgical dust and low-grade iron ore as sintering raw materials to achieve cost reduction and minimize emissions [3,4]. However, the recycling of metallurgical dust by sintering leads to the enrichment of alkali metal elements in the ironmaking process [5,6,7]. An increase in the alkali metal content of sintering raw material results in an increase in the particle size of the glass phase and a decrease in the content of low-melting-point compounds, deteriorating the compressive strength and metallurgical performance of the sinter [8,9]. Further, the alkali metal compounds in sinter are reduced to alkali metal vapor in the high-temperature zone of a blast furnace, and the alkali metal vapor is gradually absorbed on the surfaces of charges or penetrates into the furnace lining during the rising process, aggravating the pulverization of charges and the formation of scaffolds [10,11,12,13]. Therefore, to produce high-quality sinter, it is necessary to understand the influence of alkali metal compounds in different forms on the sintering mineralization process of iron ore.

Sintering dust contains a large amount of alkali metal chloride, which confirms that alkali metal elements could be removed as flue gas during sintering [14]. Therefore, over the last few decades, some metallurgists have devoted themselves to studying the sintering behavior of alkali metal compounds in different forms to elucidate the removal mechanism of alkali metal compounds in different forms. Fan et al. [15] used thermodynamic calculations and sinter pot trials to determine the reaction behavior of alkali metal compounds in different forms during sintering, and the results showed that alkali metal aluminosilicates (feldspar) were mainly converted to alkali metal oxides and less reduced to gaseous alkali metal compounds in different forms. The newly formed alkali metal oxide was transformed to alkali metal silicate/sulfate, while the gaseous alkali metal and gasified alkali metal chloride were removed as flue gas. Zhou et al. [16] studied the migration behavior of alkali metal compounds in different forms during sintering, and the results showed that the volatilization and migration of alkali metal chloride mainly occurred in the combustion layer, while the condensation and deposition of alkali metal chloride occurred in the preheating layer due to the decreasing temperature. In addition, the alkali metal chloride trapped and concentrated in the lower part of the sintering bed promoted the chlorination removal of alkali metal elements. Wang et al. [17] investigated the reduction behavior of alkali metal compounds, and the results showed that the apparent activation energy of sodium removal was higher than that of potassium removal, which indicated that potassium was more easily removed by the reduction reaction than sodium. To date, the removal mechanism of alkali metal compounds in different forms has been basically clarified, but there has been little research on the influence of alkali metal compounds in different forms on the sintering mineralization process of iron ore.

In this paper, the basic sintering characteristics of iron ore containing different quantities and forms of alkali metal compounds were determined using the micro-sintering method, allowing us to explain the influence of alkali metal compounds in different forms on the sintering mineralization process of iron ore, and the influencing mechanism was illustrated using thermodynamic analysis. However, high-purity alkali metal aluminosilicate is difficult to prepare. Hence, this paper investigated the effects of KCl/NaCl and K_2_O/Na_2_O on the basic sintering characteristics of iron ore. Furthermore, the distribution of alkali metal compounds in different forms and the microhardness of the mineral phase were detected to explain the relationship between microhardness, compressive strength, and bonding phase strength using first-principles calculations.

## 2. Materials and Methods

### 2.1. Materials

**Figure 1:**
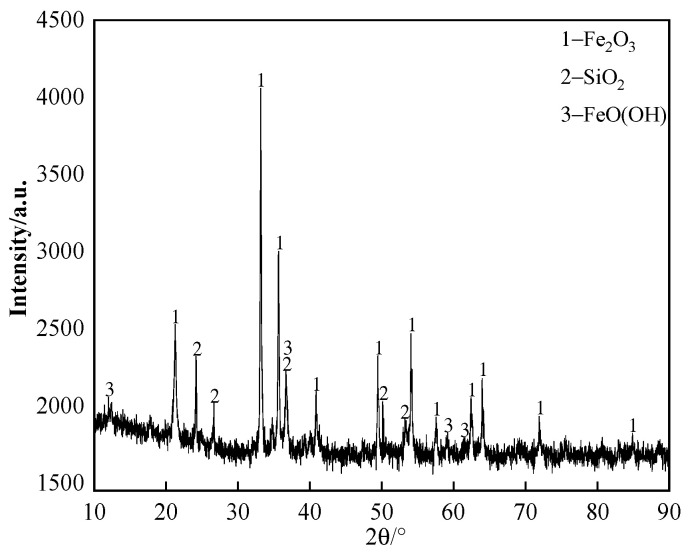
The XRD pattern of iron ore.

The chemical composition of the iron ore supplied by a domestic sintering plant is listed in Table 1; the content of K_2_O and Na_2_O in iron ore is ≤0.02%. The X-ray diffraction (XRD) pattern of the iron ore is shown in Figure 1. It can be seen that the primary iron-bearing mineral in the iron ore is hematite. The iron ore and chemical reagents were milled finely (grain size was smaller than 0.15 mm) to avoid the effect of grain size as much as possible.

Seventeen samples with different contents and forms of alkali metal compounds were investigated. The chemical compositions of samples synthesized by ore powder and chemical reagents are listed in Table 2, in which the alkali metal content changes from 0 to 4%.The samples KC1, NC1, KO1 and NO1 refer to 1%K (KCl, ≥99.5%, Damao, Tianjin, China), 1% Na (NaCl, ≥99.5%, Damao, Tianjin, China), 1%K (K_2_CO_3_, ≥99.0%, Fuchen, Tianjin, China) and 1% Na (Na_2_CO_3_, ≥99.8%, Sheng’ao, Tianjin, China), respectively.

### 2.2. Methods

#### 2.2.1. Basic Sintering Characteristics

Referring to relevant research [18,19], the micro-sintering experiment was carried out with micro-sintering equipment (BR-14AS-12, 1400 °C, Bona Heat Kiln Co., Ltd., Zhengzhou, China) to determine the basic sintering characteristics of iron ore. Wu et al. [20] proposed a detection method for the basic characteristics of high-temperature sintering. The detailed methods are shown as follows:

1. Assimilability

**Figure 2:**
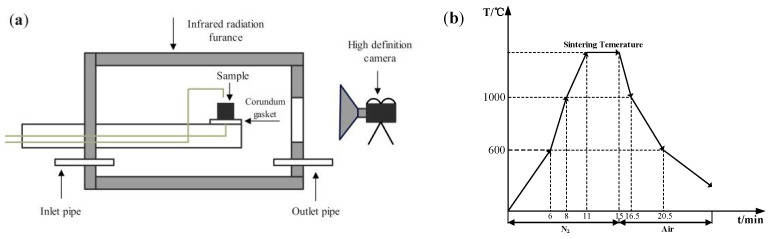
Micro-sintering equipment (**a**) and experimental conditions (**b**).

**Figure 3:**
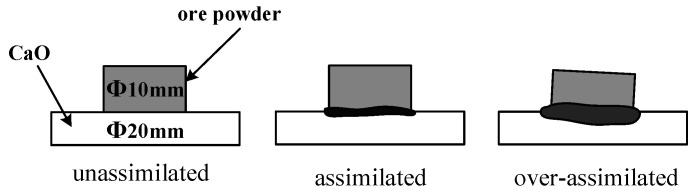
Schematic diagram of assimilation process.

In the assimilability experiment, the ore powder and alkali metal compounds were mixed thoroughly by a ball mill (MSK-SFM-12M, Kejing Star Technology Co., Ltd., Shenzhen, China). Then, 1.0 g of the mixture and 2.5 g CaO reagent (≥98.0%, Damao, Tianjin, China) were compressed under a pressure of 20 MPa for 3 min into cylindrical compacts of 10 mm and 20 mm in diameter, respectively. After the compacts were dried, the iron ore compact was put up on the CaO compact and then put into the micro-sintering equipment, which is shown in Figure 2a. The compacts were first heated to 1000 °C and then heated at 3 °C/s until the assimilated state was observed. During the heating process, 1 L/min N_2_ (99.99%) was continuously injected into the equipment and the assimilation states of iron ore at different temperatures were observed by a high-definition camera. The assimilation process of iron ore is shown in Figure 3, and the temperature at which the interface between the iron ore compact and CaO compact started to melt was recorded as the lowest assimilation temperature (LAT). The assimilability of iron ore was characterized by LAT.

2. Liquid-phase fluidity

**Figure 4:**
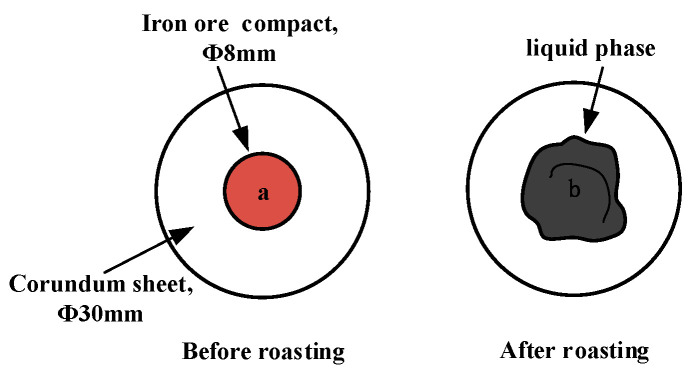
Schematic diagram of liquid-phase flow process.

In the liquid-phase fluidity experiment, the ore powder and alkali metal compounds were configured into a mixture with a basicity (CaO/SiO_2_) of 4.0 by the CaO reagent. A 0.8 g amount of mixture was compressed under a pressure of 20 MPa for 3 min into the cylindrical compact with a size of Φ8 × 5 mm. After drying, the iron ore compact was placed upon a corundum plate 30 mm in diameter and 1.5 mm in height and then put into the micro-sintering equipment. The experimental conditions are shown in Figure 2b, and the sintering temperature was set at 1280 °C. During the roasting process, 1 L/min N_2_ (heating and holding stage) and 0.5 L/min O_2_ (cooling stage) were continuously injected into the equipment. After the temperature dropped to room temperature, the corundum plate was taken out to calculate the index of liquid-phase fluidity (ILF). The liquid-phase flow process and calculation of the ILF are shown in Figure 4 and Equation (1), respectively; *a* and *b* refer to the vertical projected areas of iron ore compact and liquid phase. The liquid-phase fluidity of iron ore was characterized by the ILF.

(1) $$ILF=\frac{b-a}{a}$$

3. Bonding-phase formation characteristics

In the bonding-phase formation characteristic experiment, the ore powder and alkali metal compounds were configured into a mixture with a basicity of 2.0. A 2.0 g amount of mixture was compressed into compacts with a size of Φ15 × 8 mm. Next, dried compacts were put into a corundum crucible with a volume of 20 mL and roasted under the experimental conditions that were the same as the liquid-phase fluidity experiment. We took out the cooled sample and analyzed the copper target using a 40 kV, 40 mA X-ray diffractometer (D8 Advance A25, Bruker AXS, Karlsruhe, Germany). In step mode, with a step size of 0.01° 2 θ and a counting time of 0.15 s per step in the range of 10~90°, we worked in a step mode of 10~90 ° to determine the mineral-phase composition. We used a scanning electron microscope with an energy-dispersive spectrometer (Gemini SEM 300, Carl Zeiss AG, Oberkochen, Germany) to observe the microstructure and determine the relative content of mineral phases.

4. Strength of the bonding phase

In the experiment to determine the strength of the bonding phase, an electronic pressure tester (LD-YB-2, ±1 N, Wuzhou Special Equipment Factory, Wuzhou, China) was used to measure the compressive strength of ten sintered compacts obtained by the experiments to determine the bonding-phase formation characteristics. The strength of the bonding phase was characterized by the average compressive strength of ten sintered compacts.

#### 2.2.2. Microhardness

The microhardness testing experiment used a semi-automatic micro Vickers hardness tester (401 MAD, ±2 HV, JVC, Yokohama, Japan) to measure the microhardness of mineral phases in sintered billets, which was obtained through experiments on the formation characteristics of bonding phases. Before measuring the microhardness of the mineral phases, the sintered blocks were mixed with an epoxy resin curing agent and acrylic powder and embedded in a rubber mold with a size of Ø 24 × 12 mm, and then treated with sandpaper and a semi-automatic polishing machine (XP 810E, Guangzhou Jingying Chemical Technology Co., Ltd., Guangzhou, China). The microhardness of mineral phases is the average hardness value of 10 different points on the surface of the mineral phase. The Vickers hardness value (HV) calculation is shown in Equation (2) [21]. *F* and *d* represent a fixed experimental load (100 g) and the arithmetic average of the diagonal of the diamond indentation, respectively.

(2) $$HV=0.1891(\frac{F}{d^{2}})$$

#### 2.2.3. Thermodynamic Analysis

1. TG experiment

In the TG experiment, the mixtures configured in the bonding phase formation characteristic experiment were used. The 50 mg mixture was added to the corundum crucible, and then put into a synchronous thermal analyzer (STA449F3, Netzsch Instrument Inc., Weimar, Germany). The temperature was raised non-isothermally at 10 °C/min from room temperature to 1350 °C. Before heating up, 20 mL/min O_2_ and 50 mL/min N_2_ were injected to discharge the impurity gas, and then 20 mL/min N_2_ protection gas was injected during the heating stage.

2. Thermodynamic calculation

In the thermodynamic calculation experiment, the reaction thermodynamics of alkali metal compounds during sintering were investigated as follows: First, the primary compounds in the mixture and reaction equations were listed. Then, the reaction conditions were determined. Next, we calculated the thermodynamic properties and reactions of alkali metal compounds using the “reaction” module in FactSage 7.1. Eventually, we calculated the properties of the sintering equilibrium liquid phase of experimental mixtures containing different contents and forms of alkali metal compounds in different forms using the “Equilib” and “Viscosity” modules.

## 3. Results and Discussion

**Figure 5:**
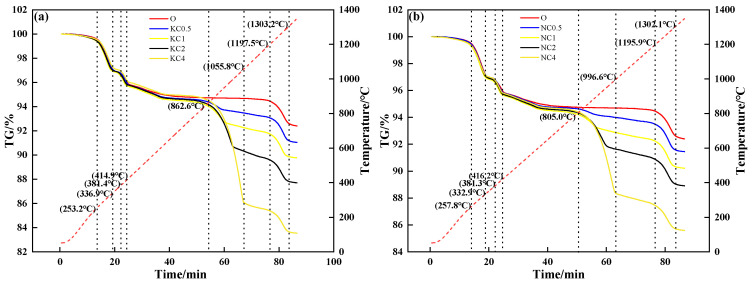

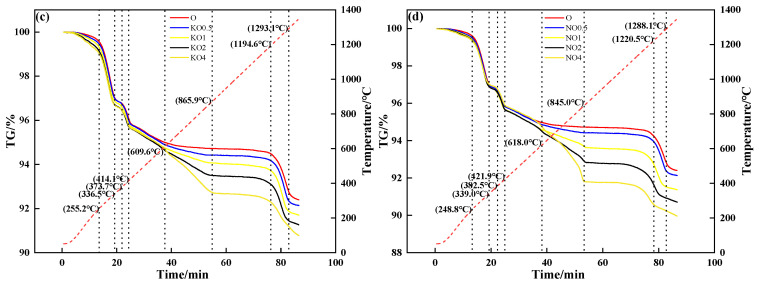
TG–time relationship of mixtures containing different contents and forms of alkali metals: (**a**) KCl; (**b**) NaCl; (**c**) K_2_CO_3_; (**d**) Na_2_CO_3_.

Figure 5 shows the TG curves of mixtures containing different contents and forms of alkali metal compounds. During the heating process, the mixture without alkali metal compounds in different forms had three mass loss stages: The first mass loss stage was 250 °C to 340 °C, which corresponded to the decomposition and volatilization of crystalline water. The second mass loss stage was 370 °C to 420 °C, which corresponded to the decomposition of Fe(OH). The third mass loss stage was 1200 °C to 1300 °C, which corresponded to the decomposition of Fe_2_O_3_. After the addition of alkali metal compounds, the fourth mass loss stage was 800 °C to 1060 °C (KCl, NaCl) and 600 °C to 870 °C (K_2_CO_3_, Na_2_CO_3_), which corresponded to the volatilization of chloride and release of CO_2_, respectively. When alkali metal content increased from 0 to 4%, Figure 5a and Figure 5b show that the TG curve decreased from 94.67% to 86.04% (KCl) and 88.36% (NaCl), respectively; Figure 5c and Figure 5d show that the TG curve decreased from 94.67% to 92.73% (K_2_CO_3_) and 91.81% (Na_2_CO_3_), respectively.

### 3.1. The Effects of Alkali Metals on the Assimilability

The results of the assimilability experiment are shown in Table 3. It can be seen from Table 3 that the lowest assimilation temperature (LAT) of iron ore without alkali metals is 1288 °C, which belongs to medium assimilability. The LAT of iron ore increased after adding alkali metal chloride while that of iron ore decreased after adding alkali metal carbonate. When alkali metal content increased to 4%, the LAT of iron ore containing KCl was highest (1300 °C), while that of iron ore containing Na_2_CO_3_ was lowest (1265 °C). The optimum assimilation temperature of iron ore is 1250–1270 °C, so the addition of alkali metal carbonate was beneficial to the assimilation of iron ore during sintering [22].

**Figure 6:**
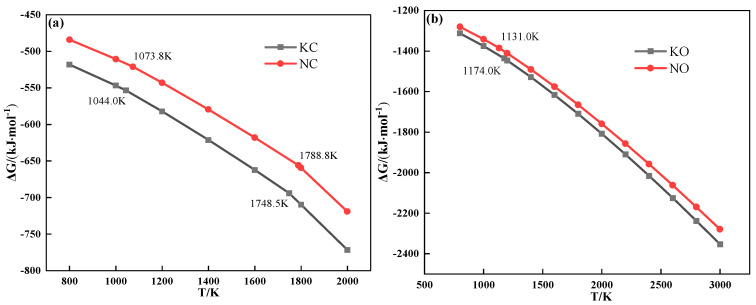
The relationship between Gibbs free energy and temperature of alkali metal chloride (**a**) and alkali metal carbonate (**b**).

The thermochemical properties of alkali metal compounds calculated by FactSage 7.1 are shown in Figure 6. It can be deduced that both alkali metal chloride and carbonate melted to form a liquid phase during sintering.

**Figure 7:**
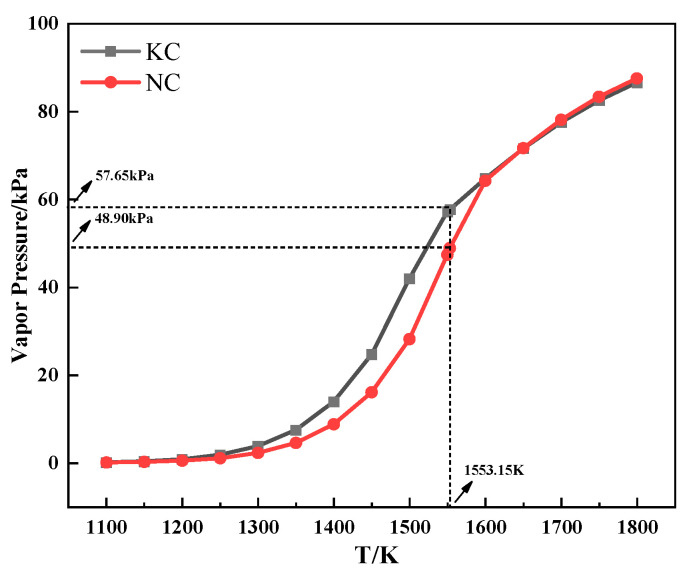
The relationship between the saturated vapor pressure and temperature of alkali metal chloride.

Figure 7 shows the relationship between the saturated vapor pressure and temperature of alkali metal chloride. As can be seen from Figure 7, the saturated vapor pressure of alkali metal chloride increased with increasing temperature. Meanwhile, the saturated vapor pressure of KCl was higher than that of NaCl when the temperature was the same and lower than 1280 °C, which indicated that KCl was more volatile during the heating process. The conclusion explains the discrepancy in the TG experiment that the TG curve of the mixture containing KCl had a larger mass loss from 800 °C to 1060 °C.

**Figure 8:**
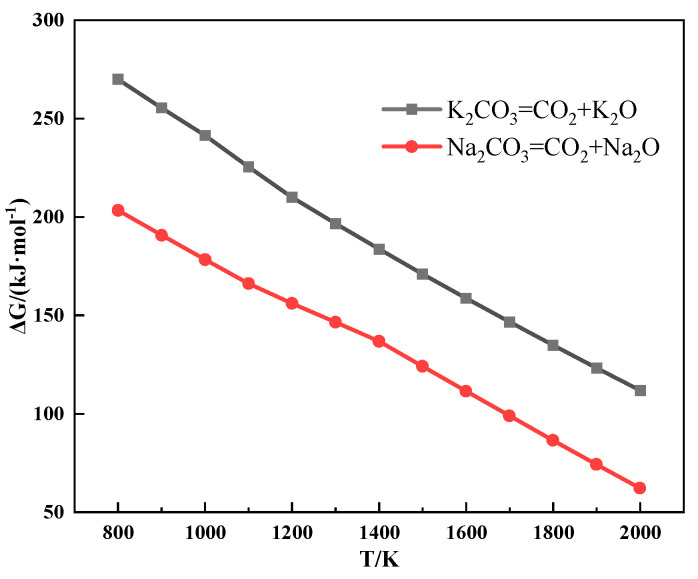
The relationship between the Gibbs free energy and temperature of the alkali metal carbonate decomposition reaction.

Figure 8 shows the thermodynamic calculation of the alkali metal carbonate decomposition reaction, which distinctly indicates that alkali metal carbonate is hard to decompose during sintering. However, the results of the TG experiment showed that CO_2_ was released from the mixture during the heating process after the addition of alkali metal carbonate, and the reaction to produce CO_2_ occurred before the alkali metal carbonate melted.

**Figure 9:**
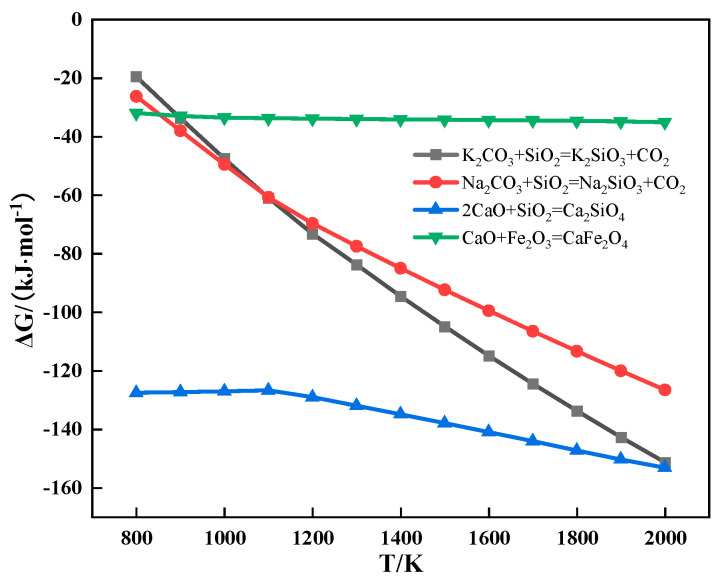
The relationship between the Gibbs free energy and temperature of partial reactions during sintering.

The relationship between the Gibbs free energy and temperature of partial reactions during sintering is shown in Figure 9. It can be seen from Figure 9 that alkali metal carbonate could react with SiO_2_ to form alkali metal silicate and CO_2_.

The alkali metal chloride gradually volatilized after melting in the sintering process, which resulted in the formation of pores at the contact interface between ore powder and CaO. The presence of these pores hindered the solid-phase diffusion of Fe^3+^ and Ca^2+^, which inhibited the formation of low-melting-point calcium ferrite. Furthermore, KCl was more volatile, so more pores were generated in the sintering process. Therefore, the LAT of iron ore increased after adding alkali metal chloride and that of iron ore containing KCl increased even more. The alkali metal carbonate reacted with SiO_2_ to form low-melting-point alkali metal silicate (≤1100 °C), which accelerated the emergence of assimilation characteristics. Meanwhile, the melting point of Na_2_CO_3_ is lower than that of K_2_CO_3_. After the alkali metal carbonate melted, it penetrated the surrounding adhesion layer and reacted with SiO_2_ to produce more alkali metal silicate. Therefore, the LAT of iron ore decreased after adding alkali metal carbonate and that of iron ore containing Na_2_CO_3_ decreased even more.

In summary, during the sintering process, the addition of alkali metal carbonate or chloride has different effects on the LAT of iron ore. Alkali metal carbonate can reduce LAT, while alkali metal chloride can increase LAT.

### 3.2. The Effects of Alkali Metals on the Liquid-Phase Fluidity

The results of the liquid-phase fluidity experiment are shown in Table 4. As can be seen from Table 4, the index of liquid-phase fluidity (ILF) of iron ore without alkali metals is 0.41. The ILF of iron ore increased with increased alkali metal content: when the alkali metal content increased to 4%, the ILF of iron ore containing Na_2_CO_3_ had the greatest increase, from 0.41 to 1.78, while that of iron ore containing KCl had the lowest increase, from 0.41 to 0.47. Therefore, adding alkali metal carbonate observably promoted the flow of the liquid phase during sintering.

**Figure 10:**
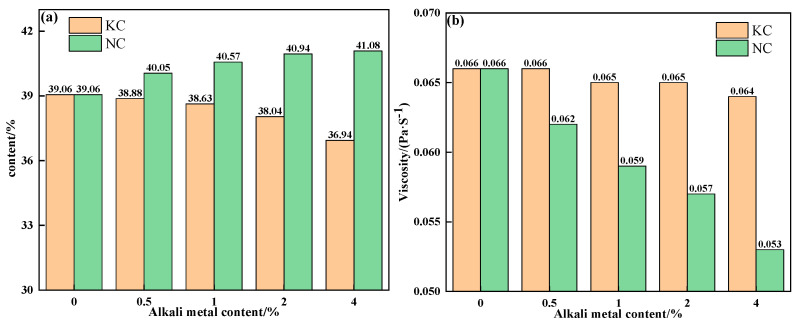
The contents (**a**) and viscosities (**b**) of the equilibrium liquid phase at 1280 °C of mixtures containing different contents of alkali metal chloride.

**Figure 11:**
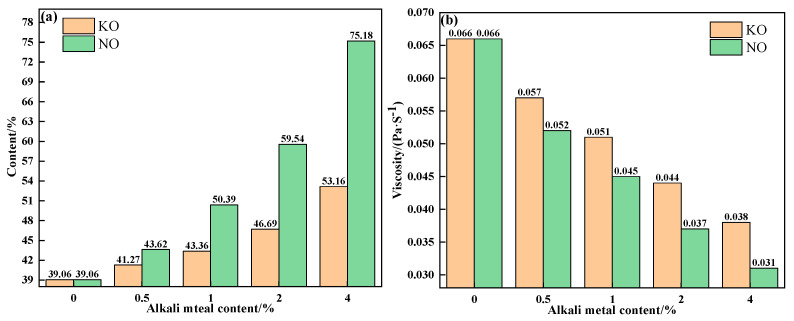
The contents (**a**) and viscosities (**b**) of the equilibrium liquid phase at 1280 °C of mixtures containing different contents of alkali metal carbonate.

To further explain the influence mechanism of different forms of alkali metals on the liquid-phase fluidity of iron ore, the properties of sintering equilibrium liquid phase of mixtures containing different contents and forms of alkali metals were calculated by FactSage 7.1, as shown in Table 5 and Table 6 and Figure 10 and Figure 11. With an increase in alkali metal content, the K_2_O/Na_2_O content in the liquid phase increased, while the viscosity of the liquid phase decreased. Research has shown that alkali metal ions decrease the viscosity of the liquid phase by destroying the Si-O net structure in the silicate liquid phase [23]. Hence, the viscosity of the liquid phase was inversely proportional to the alkali metal content in the liquid phase. In addition to KCl, adding NaCl, Na_2_CO_3_ and K_2_CO_3_ promoted the formation of the liquid phase, and Na_2_CO_3_ had the greatest effect. Therefore, under the combined influence of liquid-phase content and viscosity, the influence degree of different forms of alkali metals on the liquid-phase liquidity of iron ore from large to small was as follows: Na_2_CO_3_, K_2_CO_3_, NaCl, and KCl, which is consistent with the results of the liquid-phase fluidity experiment.

In summary, appropriately increasing the content of alkali metal carbonate or chloride can effectively improve the liquid-phase fluidity index of iron ore, and different types of alkali metals have different effects.

### 3.3. The Effects of Alkali Metals on the Bonding-Phase Formation Characteristic

**Figure 12:**
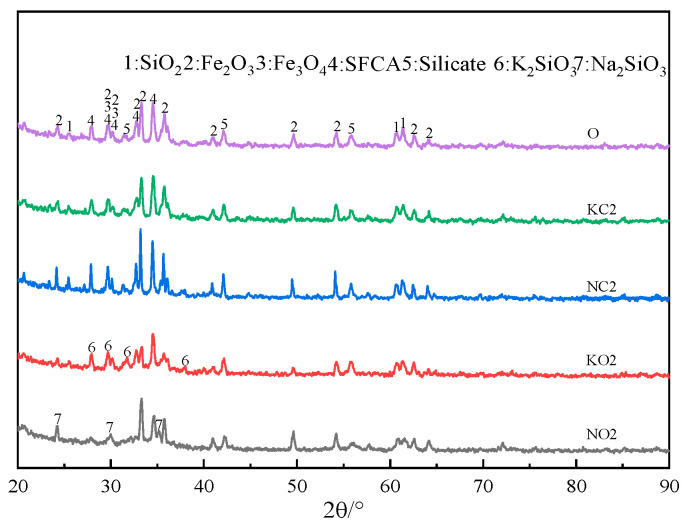
The XRD patterns of sinter after sintering iron ore containing 2% alkali metals.

The XRD patterns of sinter after sintering iron ore containing 2% alkali metals are shown in Figure 12. Figure 12 indicates that adding alkali metal carbonate in iron ore resulted in the formation of alkali metal silicate.

**Figure 13:**
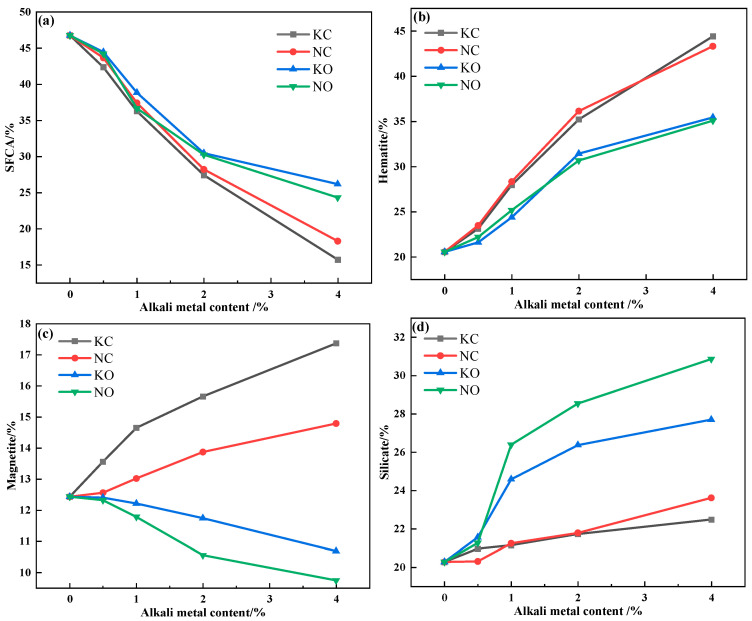
The mineral-phase content variation in sinter after sintering iron ore containing different contents and forms of alkali metals: (**a**) SFCA; (**b**) hematite; (**c**) magnetite; (**d**) silicate.

The relative area percentage of the mineral phase in the sinter obtained by Image J 1.54 h software is shown in Figure 13. It can be seen that increasing the alkali metal content decreased the SFCA content but increased the hematite and silicate content, and the addition of alkali metal chloride promoted the formation of magnetite while alkali metal carbonate inhibited the formation of magnetite.

The volatilization of alkali metal chloride was conducive to the formation of large pores, which resulted in higher porosity in the sinter. The presence of pores prevented the solid-phase reaction between iron ore (Fe_2_O_3_) and CaO, which restrained the formation of SFCA. Meanwhile, the decomposition of Fe_2_O_3_ increased the oxygen partial pressure in the reaction system, which was inconducive to the decomposition reaction. However, the increasing porosity promoted the decomposition of Fe_2_O_3_ by releasing O_2_, which decreased the content of Fe_2_O_3_ and participated in the formation of SFCA. The reaction of alkali metal carbonate and SiO_2_ resulted in the formation of a silicate liquid phase with better fluidity. On the one hand, the flowing silicate liquid phase hindered the solid-phase reaction between iron ore and CaO; on the other hand, it blocked the pores generated inside adhesion powder, which increased the Fe_2_O_3_ content and promoted the formation of SFCA. Therefore, alkali metal chloride had a greater effect on the formation of SFCA.

### 3.4. The Effects of Alkali Metals on the Strength of Bonding Phase

#### 3.4.1. The Alkali Metal Distribution and Mineral-Phase Microhardness

**Figure 14:**
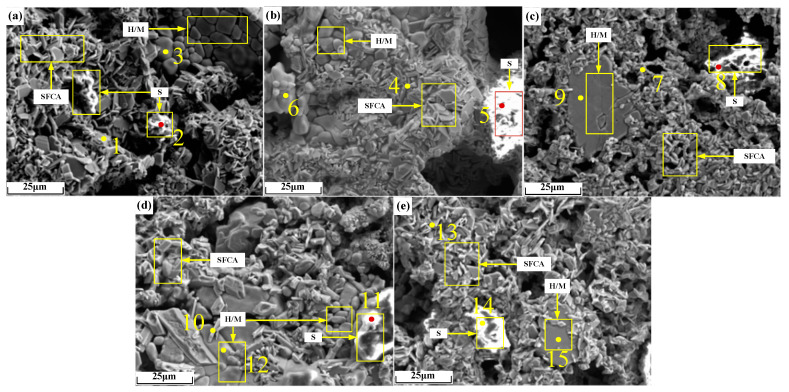
Micrographs of sinter after sintering iron ore containing 2% alkali metals: (**a**) O; (**b**) KC2; (**c**) NC2; (**d**) KO2; (**e**) NO2. H/M: hematite/magnetite.

Micrographs of sinter after sintering iron ore containing 2% alkali metals are shown in Figure 14, and the EDS analysis results of points in Figure 14 are shown in Table 7. As can be seen from Figure 14b,c, the microscopic morphology of alkali metal chloride increased by 2%, resulting in larger pores. It can be seen from Table 7 that alkali metals were found in iron-bearing minerals and silicate but to a large extent in the silicate.

During sintering, the alkali metal ions in a small amount of un-volatilized alkali metal chloride dissolved in the sintering liquid phase. During the cooling crystallization process, the silicate mineral crystals with low melting points were precipitated the last. Therefore, the alkali metal ions that finally gathered in the silicate liquid phase were enriched on the surfaces of the silicate mineral crystals. The liquid phase formed by the melting of alkali metal silicate precipitated alkali metal silicate crystals in the cooling process. Due to the high melting point, the crystals of iron-bearing minerals precipitated earlier, and the alkali metals detected in iron-bearing minerals existed in the form of solid solutions, which were formed based on the principle of charge conservation [24]. When Fe^3+^ in iron-bearing minerals was substituted by Fe^2+^ or Mg^2+^, the alkali metal ions balanced the excess negative charge by occupying a portion of the lattice space of Fe^3+^. Compared with the radius of Fe^2+^ (0.76 A), the radius of Mg^2+^ (0.65 A) is closer to that of Fe^3+^ (0.64 A). It can be seen from Table 7 that the contents of Mg and alkali metals in iron-bearing minerals in a single group of sinter showed the same change trend. Therefore, Fe^3+^ in iron-bearing minerals was more easily substituted by Mg^2+^ and alkali metal ions synchronously.

**Figure 15:**
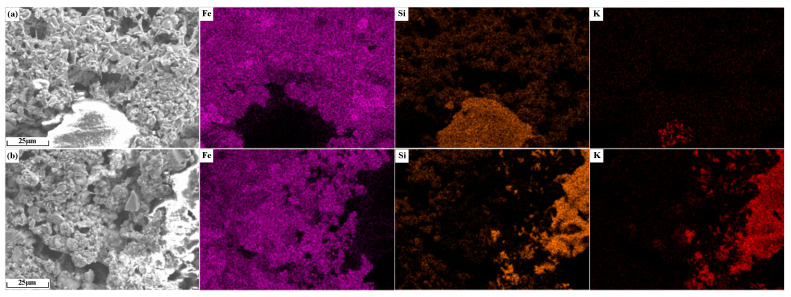
Element distribution of alkali metal-containing sinter: (**a**) KC2; (**b**) KO2.

The element distribution of potassium-containing sinter is shown in Figure 15. As shown in Figure 15a, the largest non-overlapping area of Fe and Si elements is the silicate accumulation area, and the K element is concentrated in the center of this area. As shown in Figure 15b, in the region without Fe distribution, K overlaps better with Si, which indicates that the alkali metals in sinter KO2 mainly existed in the form of silicates.

To further expose the mechanism of alkali metal deviation distribution in sinter, the Vienna ab initio simulation package (VASP 6.3.2) was used to calculate the adsorption energies of alkali metals on the mineral crystal surfaces based on first-principles calculations [25]. The Perdew–Burke–Ernzerhof (PBE) functional under general gradient approximation (GGA) was used to describe the exchange-correlation functional, and the higher accuracy of the PBE functional in describing the hydrogen bond was helpful to improve the adsorption energy [26]. The ion–electron interaction was estimated by using the projector-augmented wave model [27]. The energy cutoff for the plane wave expansion was set to 450 eV. A 3 × 3 × 1 Monkhorst Pack k-point setup was used for geometry optimization. The energy convergence for terminating the electronic self-consistent field was 10^−6^ eV/ Å, and the force convergence on each atom for geometric optimization was 10^−2^ eV/Å.

**Figure 16:**
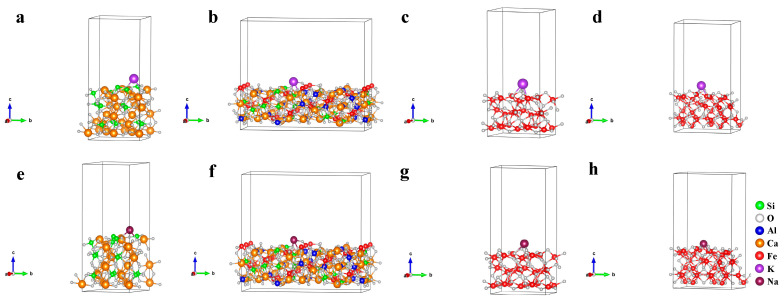
Crystalline surface adsorption models of (**a**,**e**) Ca_2_SiO_4_ (020); (**b**,**f**) SFCA (420); (**c**,**g**) Fe_2_O_3_ (104) and (**d**,**h**) Fe_3_O_4_ (220). (**a**–**d**): K adsorption, (**e**–**h**): Na adsorption.

The cell parameters before and after optimizing the mineral crystal structure are shown in Table 8; the deviations between calculation values and experience values of cell parameters are lower than 1%, belonging to a reasonable range. The crystalline surface adsorption models constructed for Fe_2_O_3_ (104), Fe_3_O_4_ (220), SFCA (420), and Ca_2_SiO_4_ (020) that corresponded to the strongest peaks in Figure 12 are shown in Figure 16.

The atomic site and cell structure of the 15 × 15 × 15 Å cell with alkali metal atoms in the central position were optimized by the VASP program under the above parameter settings. Finally, the energies of K and Na atoms were −0.1776 eV and −0.2287 eV, respectively.

**Figure 17:**
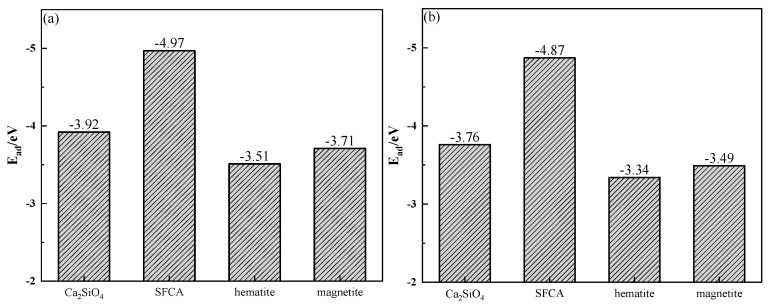
Adsorption energies of alkali metals on mineral crystal surfaces: (**a**) K; (**b**) Na.

The adsorption energy (*E_ad_*) was determined by the crystal energy (*E_alkali_*_/*crystal*_) after the adsorption of alkali metal, the crystal surface energy (*E_crystal_*) before the adsorption of alkali metal, and the atomic energy of alkali metal (*E_alkali_*). The calculation of *E_ad_* is shown in Equation (3) [28], and the calculation results are shown in Figure 17. Figure 17 shows that the adsorption of alkali metals on the SFCA crystal surface released energy the most, which meant that the SFCA crystal adsorbing alkali metals was more stable. Therefore, among the iron-bearing minerals, SFCA contained more alkali metal-bearing solid solutions.

(3) $$E_{ad}=E_{alkali/cryatal}-(E_{cryatal}+E_{alkali})$$

Microhardness detection results are shown in Table 9. As can be seen from Table 9, with increasing alkali metal content, the microhardness of iron-bearing minerals decreased, and that of SFCA decreased the most, while that of silicate had no obvious variation.

As shown in Table 7 and Table 9, it can be deduced that the microhardness values of minerals were inversely proportional to the amount of an alkali metal-bearing solid solution in the minerals. Hence, the formation of alkali metal-bearing solid solution resulted in the deterioration of the micromechanical properties of iron-bearing minerals.

#### 3.4.2. Compressive Strength

**Figure 18:**
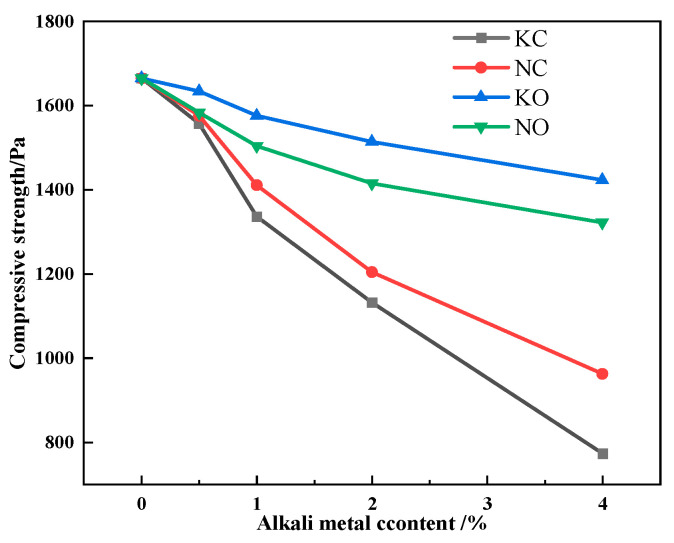
The effects of different forms of alkali metals on the compressive strength.

The results of compressive strength detection are shown in Figure 18. Figure 18 shows that the compressive strength of the sinter decreased as the alkali metal content increased, and the compressive strength of the sinter after sintering iron ore containing KCl decreased more significantly.

Since the microhardness of iron-bearing minerals in the alkali metal-bearing sinter worsened, the compressive strength of the sinter worsened accordingly. Nevertheless, Table 8 indicates that alkali metal carbonate had a greater effect on the microhardness of the mineral, while alkali metal chloride had a greater effect on the compressive strength of the sinter. Meanwhile, alkali metals had the same effect on the SFCA bonding-phase formation characteristic and compressive strength. Therefore, the formation of a solid solution and the decrease in SFCA content worsened the bonding-phase strength, which directly brought about the decreasing compressive strength of the sinter.

## 4. Conclusions

In this study, the basic sintering characteristics of iron ore were measured by the micro-sintering method, and the affecting mechanism of KCl, NaCl, K_2_CO_3_ and Na_2_CO_3_ on the basic sintering characteristics of iron ore was clarified by thermodynamic analysis and first-principles calculations. The following conclusions can be made:

1. The LAT and ILF of iron ore increased with increasing contents of KCl and NaCl; increasing the contents of K_2_CO_3_ and Na_2_CO_3_ decreased the LAT but increased the ILF of iron ore. The volatilization of KCl/NaCl led to the formation of pores, which restrained the formation of low-melting-point calcium ferrite. The addition of K_2_CO_3_/Na_2_CO_3_ promoted the formation of low-melting-point alkali metal silicate.
2. The pores caused by the volatilization of KCl/NaCl hindered the diffusion of Fe^3+^ and Ca^2+^, which suppressed the formation of SFCA. K_2_CO_3_/Na_2_CO_3_ reacted with SiO_2_ to form K_2_SiO_3_/Na_2_SiO_3_, which melted to form a liquid phase with better fluidity. The flowing liquid phase intervened in the solid-phase reaction between iron ore and CaO, which decreased the SFCA content in the sinter.
3. The alkali metals were mainly concentrated in silicate, and less distributed in iron-bearing minerals in the form of a solid solution. Among the iron-bearing minerals, the adsorption energies of alkali metals on the SFCA crystal surface were the lowest. The formation of a solid solution decreased the microhardness of the SFCA bonding phase, and the decrease in the microhardness and content of the SFCA bonding phase resulted in a decrease in bonding-phase strength.
4. The iron ore with high alkali metal content presented poor basic sintering characteristics during sintering. Therefore, both removing the alkali metals and ore blending optimization based on basic sintering characteristics are not only good approaches for recovering metallurgical dust but can also play a role in sintering industry applications by reducing costs and improving efficiency, which are future research directions.

## Figures and Tables

**Table 1:** Chemical composition of iron ore (%).

TFe	CaO	SiO_2_	Al_2_O_3_	MgO	K_2_O	Na_2_O
61.28	0.17	5.27	2.69	0.26	0.02	--

**Table 2:** Chemical compositions of iron ore containing different contents and forms of alkali metal compounds (%).

Samples	TFe	CaO	SiO_2_	Al_2_O_3_	MgO	KCl	NaCl	K_2_CO_3_	Na_2_CO_3_
O	61.28	0.17	5.27	2.69	0.26	0.00	--	--	--
KC0.5	60.69	0.17	5.22	2.66	0.26	0.96	--	--	--
KC1	60.11	0.17	5.17	2.64	0.26	1.91	--	--	--
KC2	58.94	0.16	5.07	2.59	0.25	3.82	--	--	--
KC4	56.60	0.16	4.87	2.48	0.24	7.63	--	--	--
NC0.5	60.50	0.17	5.20	2.66	0.26	--	1.28	--	--
NC1	59.72	0.17	5.14	2.62	0.25	--	2.55	--	--
NC2	58.16	0.16	5.00	2.55	0.25	--	5.09	--	--
NC4	55.05	0.15	4.73	2.42	0.23	--	10.17	--	--
KO0.5	60.73	0.17	5.22	2.67	0.26	--	--	0.89	--
KO1	60.20	0.17	5.18	2.64	0.26	--	--	1.77	--
KO2	59.11	0.16	5.08	2.59	0.25	--	--	3.54	--
KO4	56.95	0.16	4.90	2.50	0.24	--	--	7.07	--
NO0.5	60.57	0.17	5.21	2.66	0.26	--	--	--	1.16
NO1	59.86	0.17	5.15	2.63	0.25	--	--	--	2.31
NO2	58.45	0.16	5.03	2.57	0.25	--	--	--	4.61
NO4	55.63	0.15	4.78	2.44	0.24	--	--	--	9.22

**Table 3:** The effects of different forms of alkali metals on the LAT.

Sample	KC0	KC0.5	KC1	KC2	KC4	NC0	NC0.5	NC1	NC2	NC4
LAT/°C	1288	1289	1293	1295	1300	1288	1288	1290	1293	1295
Sample	KO0	KO0.5	KO1	KO2	KO4	NO0	NO0.1	NO1	NO2	NO4
LAT/°C	1288	1285	1281	1278	1270	1288	1284	1277	1274	1265

**Table 4:** The effects of different forms of alkali metals on the ILF.

Sample	KC0	KC0.5	KC1	KC2	KC4	NC0	NC0.5	NC1	NC2	NC4
ILF	0.41	0.4	0.44	0.45	0.47	0.41	0.43	0.46	0.52	0.74
Sample	KO0	KO0.5	KO1	KO2	KO4	NO0	NO0.1	NO1	NO2	NO4
ILF	0.41	0.48	0.54	0.77	1.09	0.41	0.56	0.82	1.17	1.78

**Table 5:** The compositions of the equilibrium liquid phase at 1280 °C of mixtures containing different contents of alkali metal chloride (%).

Samples	Fe_2_O_3_	Al_2_O_3_	SiO_2_	CaO	FeO	MgO	K_2_O	Na_2_O
O	57.839	4.085	11.064	22.152	4.730	0.131	0.000	0.000
KC0.5	57.971	4.039	11.019	22.077	4.764	0.125	0.004	0.000
KC1	58.021	3.996	11.019	22.048	4.786	0.124	0.006	0.000
KC2	58.075	3.958	11.019	22.009	4.807	0.121	0.010	0.000
KC4	58.241	3.904	10.970	21.901	4.855	0.113	0.016	0.000
NC0.5	58.828	3.891	10.687	21.358	4.946	0.124	0.000	0.166
NC1	59.458	3.774	10.431	20.874	5.077	0.122	0.000	0.264
NC2	60.248	3.607	10.118	20.206	5.290	0.115	0.000	0.416
NC4	61.380	3.372	9.643	19.232	5.619	0.105	0.000	0.650

**Table 6:** The compositions of the equilibrium liquid phase at 1280 °C of mixtures containing different contents of alkali metal carbonate (%).

Samples	Fe_2_O_3_	Al_2_O_3_	SiO_2_	CaO	FeO	MgO	K_2_O	Na_2_O
O	57.839	4.085	11.064	22.152	4.730	0.131	0.000	0.000
KO0.5	60.222	3.085	10.414	20.806	5.220	0.120	0.134	0.000
KO1	61.952	2.229	9.835	19.692	5.788	0.115	0.389	0.000
KO2	63.758	1.232	8.994	18.007	6.436	0.111	1.462	0.000
KO4	65.752	0.953	7.241	14.483	6.780	0.103	4.687	0.000
NO0.5	61.572	3.255	9.610	19.050	5.652	0.123	0.000	0.738
NO1	64.454	2.978	8.413	16.862	5.756	0.120	0.000	1.418
NO2	67.792	2.624	6.984	13.998	5.874	0.119	0.000	2.610
NO4	71.110	2.284	5.312	10.636	6.051	0.143	0.000	4.464

**Table 7:** EDS analysis results (at%).

Points	Fe	Ca	Si	Al	Mg	O	Na	K	Minerals
1	26.56	8.11	2.76	1.23	0.68	60.66	--	--	SFCA
2	3.37	16.26	12.34	0.12	0.16	67.75	--	--	Silicate
3	38.23	0.38	0.07	0.18	0.17	60.97	--	--	H/M
4	29.96	7.84	2.38	1.28	0.99	57.47	--	0.08	SFCA
5	5.60	16.33	8.68	9.97	0.26	58.82	--	0.34	Silicate
6	44.10	0.31	0.07	0.16	0.14	55.18	--	0.04	H/M
7	25.37	7.20	3.61	2.14	1.44	60.09	0.15	--	SFCA
8	5.54	14.39	9.67	8.37	0.82	60.34	0.87	--	Silicate
9	41.38	0.19	0.12	0.25	0.20	57.76	0.10	--	H/M
10	25.25	9.36	3.50	0.94	1.13	59.10	--	0.72	SFCA
11	4.70	0.52	15.87	0.44	0.33	55.32	--	22.82	Silicate
12	39.04	0.91	0.15	0.06	0.17	59.47	--	0.20	H/M
13	25.19	6.58	2.68	1.58	0.99	62.54	0.44	--	SFCA
14	6.71	20.01	15.75	0.31	0.15	55.85	1.22	--	Silicate
15	37.18	0.18	0.12	0.33	0.27	61.71	0.21	--	H/M

**Table 8:** Optimized cell parameters of mineral crystals (Å).

Crystals		a	b	c
Ca_2_SiO_4_	Calc	6.784	5.494	9.261
	Exp	6.737	5.481	9.174
	Error	0.69%	0.24%	0.94%
SFCA	Calc	10.080	10.660	9.110
	Exp	10.050	10.558	9.069
	Error	0.30%	0.96%	0.45%
Fe_2_O_3_	Calc	5.032	5.032	13.733
	Exp	5.024	5.024	13.693
	Error	0.16%	0.16%	0.29%
Fe_3_O_4_	Calc	8.399	8.399	8.399
	Exp	8.389	8.389	8.389
	Error	0.12%	0.12%	0.12%

**Table 9:** Microhardness values of mineral phases in sinter after sintering iron ore containing different contents and forms of alkali metals (HV).

Samples	Silicate	SFCA	Hematite	Magnetite
O	404.3	891.4	1056.5	775.2
KC0.5	405.2	867.1	1055.2	763.4
KC1	410.3	839.1	1049.4	754.2
KC2	408.2	802.6	1027.9	732.7
KC4	395.6	787.7	1006.7	703.2
NC0.5	402.5	869.4	1050.5	766.8
NC1	403.1	835.1	1028.2	740.3
NC2	407.3	787.2	988.0	701.8
NC4	411.2	769.2	968.7	682.9
KO0.5	410.8	839.6	1028.3	745.7
KO1	405.7	808.5	986.6	701.6
KO2	392.1	756.7	966.4	654.5
KO4	394.6	685.5	933.9	628.7
NO0.5	399.2	846.1	1039.7	754.7
NO1	411.5	801.2	1010.4	721.4
NO2	407.6	762.7	982.3	694.8
NO4	403.2	725.1	966.4	667.9

## Data Availability

The original contributions presented in this study are included in the article. Further inquiries can be directed to the corresponding author.
